# Longitudinal Retinal and Choroidal Image Analysis in a Set of Monozygotic Twins

**DOI:** 10.7759/cureus.54557

**Published:** 2024-02-20

**Authors:** Angela M Hemesath, Justin P Ma, Bryce W Polascik, Dilraj Grewal, Sharon Fekrat

**Affiliations:** 1 Department of Ophthalmology, Duke University School of Medicine, Durham, USA; 2 iMIND (Eye Multimodal Imaging in Neurodegenerative Disease) Study Group, Duke University School of Medicine, Durham, USA

**Keywords:** optical coherence tomography, case study, retina, longitudinal study, monozygotic twins

## Abstract

We analyzed multimodal retinal and choroidal imaging, including optical coherence tomography (OCT) and OCT angiography (OCTA), to assess differences and characterize variations in the retinal and choroidal structure and microvasculature between healthy monozygotic twins without ocular or systemic pathology over a five-year period. Retinal imaging of both subjects revealed normal age-related changes. There was up to an 11% difference in OCT and OCTA variables within the subjects, both at baseline and at five years, and there was up to an 18% difference in OCT and OCTA parameters between the subjects for both time points. Larger changes in subfoveal choroidal thickness and foveal avascular zone area were observed. Our observations suggest that the parafoveal superficial capillary plexus, choroidal vascularity index, central subfield thickness, retinal nerve fiber layer thickness, and ganglion cell-inner plexiform layer thickness may be more heavily influenced by genetic, rather than environmental, factors. In contrast, subfoveal choroidal thickness and the foveal avascular zone area may be more heavily influenced by environmental factors. The environmental impact on retinal and choroidal structure and microvasculature is increasingly important to characterize, as such imaging parameters are being explored as potential biomarkers of systemic disease. These differences, as seen in these identical twin subjects, may be important considerations in supporting the security of biometric identifiers.

## Introduction

Twin studies date back to 1811 and are a cornerstone of genetic and epigenetic research [1]. Because they are assumed to have identical genetic data, monozygotic twin studies are an especially powerful tool to better understand the interplay between environmental and genetic effects on disease. In ophthalmology, twin studies have been instrumental in defining genetic and environmental contributions to ocular diseases, such as age-related macular degeneration and retinopathy of prematurity, among others [2,3]. A growing body of research has illustrated that retinal imaging parameters are highly associated with the progression of both heritable and acquired diseases [4,5].

Similarly, biometric research has shown that optical coherence tomography (OCT) and OCT angiography (OCTA) can be alternate means of personal identification, potentially replacing or functioning as an adjunct to fundus photography [6]. Retinal imaging has been established as the most robust method for biometric authentication and has been used in high-security facilities [7]. In part, this is due to Simon and Goldstein’s study in 1935, which revealed that the retinal vasculature differs among individuals, including identical twins [8]. However, precisely how retinal imaging metrics differ between identical twins needs further study. Moreover, retinal vasculature and microvasculature may be subject to the consequences of disease, trauma, or therapeutic intervention.

There are limited studies of longitudinal changes in OCT and OCTA parameters, particularly in monozygotic twins. Understanding the effect of time on retinal and choroidal parameters in healthy monozygotic twins with no ocular or systemic disease presents a unique opportunity to better document individual and age-related changes in these emerging biometric parameters. Herein, we characterize the longitudinal differences in retinal and choroidal imaging between each twin in one set of monozygotes, as well as compare longitudinal measurements from each imaging modality, using a multimodal imaging approach with OCT and OCTA.

## Case presentation

This study was approved by the Duke Health Institutional Review Board (Pro00082598). The Health Insurance Portability and Accountability Act (HIPPA) of 1996 and all tenets of the Declaration of Helsinki were followed. Participants signed written informed consent prior to participation.

Participants

Participants were a pair of monozygotic, twin, 44-year-old adult males who expressed interest in participation as controls with normal cognition following receipt of the Eye Multimodal Imaging in Neurodegenerative Disease (iMIND) recruitment flyer. Exclusion criteria included any medical conditions, such as diabetes, hypertension, cardiovascular disease, glaucoma, any retinal or optic nerve pathology, prior vitreoretinal procedures, and corrected distance Snellen visual acuity (VA) less than 20/40. Subjects underwent two visits, spaced five years apart. Subjects underwent a Mini-Mental State Exam (MMSE) at both visits. Ultra-widefield (UWF) fundus photography was captured using California scanning laser ophthalmoscopy (Optos, Marlborough, MA, USA) at each visit to evaluate for retinal, choroidal, or optic nerve pathology. Images were reviewed and evaluated by trained graders (AMH, JM) and suspicious pathology was reviewed by field experts (DSG, SF).

Image acquisition

At both study visits five years apart, undilated OCT and OCTA images were obtained with the Zeiss Cirrus HD-5000 Spectral-Domain OCT with AngioPlex (Carl Zeiss Meditec, Dublin, CA, USA). For each eye at each visit, OCT image acquisition included a 200x200 μm optic disc cube, HD 21-line with enhanced depth imaging, and 512x128 μm macular cube scan. All images were reviewed by trained study personnel for quality. Any OCT or OCTA images with less than 7/10 signal strength, focal signal loss, or artifacts were excluded. Zeiss software-calculated parameters included average retinal nerve fiber layer (RNFL) thickness, central subfield thickness (CST), and ganglion cell-inner plexiform layer (GC-IPL) thickness. Subfoveal choroidal thickness (SFCT) was manually measured by a trained iMIND grader (AMH) and confirmed by an experienced retina specialist and image grader (DSG).

Measurements of superficial capillary plexus (SCP) perfusion density (PD) and vessel density (VD) were automatically quantified within the Early Treatment Diabetic Retinopathy Study (ETDRS) 3x3 mm and 6x6 mm grid overlaid onto macular OCTA scans centered on the fovea (AngioPlex Vessel Map, Carl Zeiss Meditec, version 10.0.0.12787). Another Zeiss software-generated parameter collected included the SCP foveal avascular zone (FAZ) area measured on the 3x3 mm scan. VD and PD measurements were measured in the 6 mm circle, 6 mm outer ring, 6 mm inner ring, 3 mm circle, and 3 mm ring.

**Figure 1:**
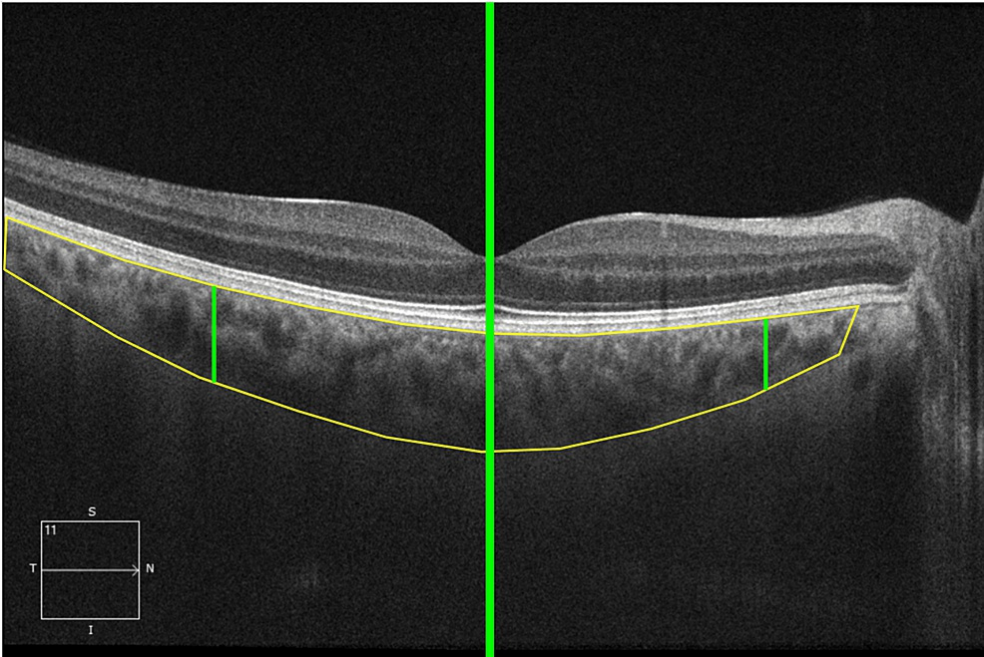
Region of interest outline in the COIN software An example of a manual outline of the choroid using the Comprehensive Ocular Imaging Network (COIN) online portal (www.ocularimaging.net, v06.26) software. The 1500 μm region of interest (between the right-most and left-most green lines) was centered under the fovea (thick middle green line).

The choroidal vascularity index (CVI), the ratio of choroid luminal area and total choroidal area, was calculated on the HD 21-line enhanced depth imaging (EDI) foveal scan using the Comprehensive Ocular Imaging Network (COIN) online portal (www.ocularimaging.net, v06.26) and its built-in algorithm [9]. The region of interest in the choroid layer, defined as the area between the outer border of the hyperreflective retinal pigment epithelium and the sclerochoroidal junction, was manually outlined by a trained iMIND grader, confirmed by an experienced retina specialist, and comprised a horizontal area 1500 µm wide, centered on the fovea. The total choroidal area (TCA) within the region was automatically calculated and the luminal area (LA), defined as the area of dark pixels in the choroid, was also determined. The COIN software automatically calculated the CVI by dividing LA by PCA (Figure 1).

Statistical analysis

The main outcomes measured were the percent change over five years, velocity in OCT and OCTA parameters at five years compared to baseline, and the absolute percent difference in parameters between the subjects. For the absolute percent difference between the subjects, the right eye of twin one was compared to the right eye of twin two, and the left eye of twin one was compared to the left eye of twin two. The absolute difference between twin parameters was normalized in order to compare changes between OCT and OCTA parameters. Twin one was always used as the denominator for consistency. 

Percent change over the five-year follow-up was calculated as below:

\[\frac{(follow\mbox{-}up\mbox{ }parameter)-(baseline\mbox{ }parameter)}{(baseline\mbox{ }parameter)} \times 100\]

Velocity over the five-year follow-up was calculated as below:

\[\frac{(follow\mbox{-}up\mbox{ }parameter)-(baseline\mbox{ }parameter)}{(time\mbox{ }in\mbox{ }years)}\]

The absolute percent difference in parameters between the subjects was calculated as below:

\[|\frac{((Twin\mbox{ }one\mbox{ }parameter)-(Twin\mbox{ }two\mbox{ }parameter)}{(Twin\mbox{ }one\mbox{ }parameter)}| \times 100\]

Results

**Figure 2:**
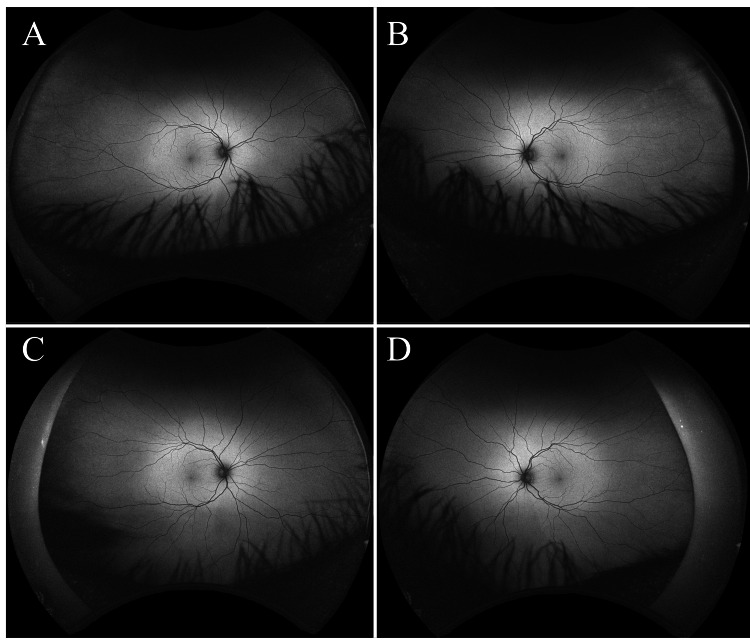
Autofluorescence ultra-widefield fundus images at age 44 The top row shows autofluorescence of the right eye (A) and left eye (B) of twin one at baseline. The bottom shows autofluorescence images of the right eye (C) and left eye (B) of twin two at baseline. Retinal vasculature branching patterns were not identical between the twins. There was no evidence of retinal, choroidal, or optic nerve pathology. Eyelid and eyelash artifacts were present.

**Figure 3:**
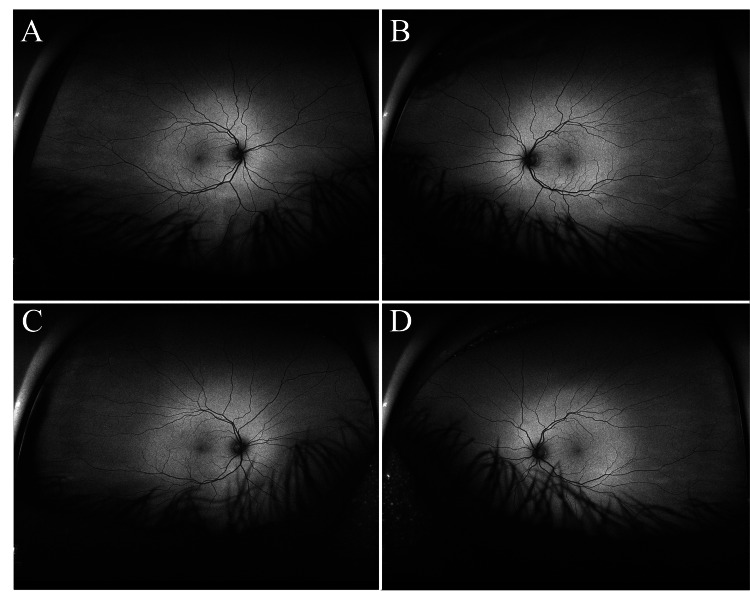
Autofluorescence ultra-widefield fundus images five years later at age 49 The top row shows autofluorescence of the right eye (A) and left eye (B) of twin one at follow-up. The bottom show shows autofluorescence images of the right eye (C) and left eye (B) of twin two at follow-up. There was no evidence of retinal, choroidal, or optic nerve pathology. Eyelid and eyelash artifacts were present.

A pair of 44-year-old, healthy, male monozygotic twins volunteered to undergo retinal and choroidal imaging at baseline and five years later at age 49. The participants shared the same environment in childhood and adolescence, the same undergraduate and graduate institutions, and the same occupation, working at the same institution. At baseline, both were emmetropic, and phakic with clear lenses, and had 20/20 uncorrected distance visual acuity in each eye. Mini-Mental State Examination (MMSE) scores for both twins were normal (30/30) at both baseline and the five-year visit. Neither was taking any medication, had ever smoked, or had ever had hypertension, diabetes, or other conditions that could affect the retina and possibly preclude comparison. At year five, both remained at their baseline ocular state, and neither had any changes in health history. Ultra-widefield (California, Optos) imaging showed no retinal, choroidal, or optic nerve pathology at baseline or five years later (Figure 2 and Figure 3). Additionally, retinal vasculature branching was not phenotypically identical in the subjects.

Image quality at baseline and five years later was superb, with 10/10 signal strength for all OCT and OCTA images.

Comparison of Baseline and Five-Year Follow-Up Retinal Imaging Parameters

At the five-year follow-up, OCT parameters between the twins had an absolute percent difference of less than 11%. CST, GC-IPL thickness, and RNFL thickness had a change of less than 5%. Subfoveal choroidal thickness decreased in both twins by up to 10.9%. CVI decreased in both eyes of both twins by up to 3.2%.

Five years later, superficial capillary plexus perfusion density (PD) and vessel density (VD) OCTA parameters decreased by a maximum of 6.9% in the twins. FAZ area increased at five years for both eyes of both subjects, with twin one increasing by 5.9% right eye and 11.1% left eye. Twin two FAZ area increased by 5% right eye and 10.5% left eye. Results are shown in Table 1.

**Table 1:** Within-subject comparison of OCT and OCTA data CST = central subfield thickness, CVI = choroidal vascularity index, FAZ = foveal avascular zone, GC-IPL= ganglion cell-inner plexiform layer, OCT = optical coherence tomography, OCTA = OCT angiography, OD = right eye, OS = left eye, PD = perfusion density (unitless), RNFL = retina nerve fiber layer, VD = vessel density

		Twin one		Twin two	
		percent change (%)		percent change (%)	
OCT variables		OD	OS	OD	OS
	CST (µm)	4.7	0.0	3.0	2.6
	GC-IPL thickness (µm)	2.5	0.0	1.2	1.2
	RNFL thickness (µm)	0	1.2	3.4	1.1
	Subfoveal choroidal thickness (µm)	-1.6	-9.7	-7.2	-10.9
	CVI (%)	-3.2	-1.3	-0.3	-1.2
OCTA variables					
	FAZ area (mm^2^)	5.9	11.1	5.0	10.5
	PD (3 mm circle)	-1.0	-5.2	-2.5	-6.9
	PD (3 mm ring)	-1.0	-6.8	-1.9	-6.3
	VD (3 mm circle) (mm^-1^)	-1.8	-5.5	-3.1	-6.6
	VD (3 mm ring) (mm^-1^)	-1.7	-5.3	-2.6	-5.9
	PD (6 mm circle)	-2.6	-0.4	-4.1	-5.7
	PD (6 mm inner ring)	-5.5	-1.7	-4.4	-4.6
	PD (6 mm outer ring)	-1.5	0.0	-3.8	-6.1
	VD (6 mm circle) (mm^-1^)	-2.1	-0.5	-3.6	-5.2
	VD (6 mm inner ring) (mm^-1^)	-4.1	-2.1	-4.0	-5.0
	VD (6 mm outer ring) (mm^-1^)	-1.6	-0.5	-3.1	-5.2

Comparison of Parameter Velocity

For both subjects, CST, GC-IPL thickness, RNFL thickness, and FAZ area increased in both eyes at five years. PD and VD decreased bilaterally for both subjects in the ETDRS 3x3 mm circle and ring, and 6x6 mm circle, inner ring, and outer ring. SFCT decreased in both eyes of both subjects. CVI decreased in both eyes of twin one and decreased in the left eye of twin two. However, twin two right eye CVI increased by 0.036%/year. OCT and OCTA parameter velocities are demonstrated in Table 2.

**Table 2:** Velocity of OCT and OCTA metrics of each twin from baseline to five years CST = central subfield thickness, CVI = choroidal vascularity index, FAZ = foveal avascular zone, GC-IPL= ganglion cell-inner plexiform layer, OCT = optical coherence tomography, OCTA = OCT angiography, OD = right eye, OS = left eye, PD = perfusion density (unitless), RNFL = retina nerve fiber layer, VD = vessel density

		Twin one		Twin two	
		Velocity (units/year)		Velocity (units/year)	
OCT variables		OD	OS	OD	OS
	CST (µm)	2.4	0.0	1.6	1.4
	GC-IPL thickness (µm)	0.4	0.0	0.2	0.2
	RNFL thickness (µm)	0.0	0.2	0.6	0.2
	Subfoveal choroidal thickness (µm)	-8.2	-10	-5.4	-9.2
	CVI (%)	-0.172	-0.084	0.036	-0.158
OCTA variables					
	FAZ area (mm^2^)	0.002	0.004	0.002	0.004
	PD (3 mm circle)	-0.0008	-0.004	-0.002	-0.0054
	PD (3 mm ring)	-0.0008	-0.0056	-0.0016	-0.0052
	VD (3 mm circle) (mm^-1^)	-0.08	-0.24	-0.14	-0.3
	VD (3 mm ring) (mm^-1^)	-0.08	-0.24	-0.12	-0.28
	PD (6 mm circle)	-0.0024	-0.0004	-0.0038	-0.0054
	PD (6 mm inner ring)	-0.0052	-0.0016	-0.0042	-0.0044
	PD (6 mm outer ring)	-0.0014	0	-0.0036	-0.0058
	VD (6 mm circle) (mm^-1^)	-0.08	-0.02	-0.14	-0.2
	VD (6 mm inner ring) (mm^-1^)	-0.16	-0.08	-0.16	-0.2
	VD (6 mm outer ring) (mm^-1^)	-0.06	-0.02	-0.12	-0.2

Comparison Between Twin One and Twin Two

When comparing twin one to twin two, baseline parameters of CST, GC-IPL thickness, RNFL thickness, subfoveal choroidal thickness, and CVI had an absolute percent difference of < 6% in both eyes. At five years, the same OCT parameters had an absolute percent difference of up to 8.1%, with the largest differences observed in subfoveal choroidal thickness (8.1%) right eye and CST (1.6%) left eye.

At baseline, OCTA parameters of VD and PD in the 3 mm circle and ring and 6 mm circle, inner ring, and outer ring had an absolute percent difference of < 5% in both eyes between the twins. The absolute percent difference in the SCP FAZ area was 17.6% right eye and 5.6% left eye between the twins at baseline.

At five years, all PD and VD parameters in both eyes of the twins had an absolute percent difference of < 4%. The FAZ area had an absolute percent difference of 14.3% right eye and 4.8% left eye. The absolute percent differences between subjects are shown in Table 3. 

**Table 3:** Absolute percent difference in OCT and OCTA metrics between twins at baseline and five years later CST = central subfield thickness, CVI = choroidal vascularity index, FAZ = foveal avascular zone, GC-IPL= ganglion cell-inner plexiform layer, OCT = optical coherence tomography, OCTA = OCT angiography, OD = right eye, OS = left eye, PD = perfusion density (unitless), RNFL = retina nerve fiber layer, VD = vessel density

		Baseline		five years	
		absolute percent difference (%)		absolute percent difference (%)	
OCT variables		OD	OS	OD	OS
	CST (µm)	5	3.9	3.3	6.2
	GC-IPL thickness (µm)	1.3	1.3	0.0	2.4
	RNFL thickness (µm)	2.2	5.8	1.1	5.4
	Subfoveal choroidal thickness (µm)	1.8	0.24	8.1	1.6
	CVI (%)	2.8	0.25	0.6	0.1
OCTA variables					
	FAZ area (mm^2^)	17.6	5.6	14.3	4.8
	PD (3 mm circle)	1.5	2.6	0	0.8
	PD (3 mm ring)	1.0	0.7	0	1.3
	VD (3 mm circle) (mm^-1^)	0.4	3.2	1.8	1.9
	VD (3 mm ring) (mm^-1^)	0.8	3.5	1.8	2.7
	PD (6 mm circle)	0.9	2.8	0.67	2.7
	PD (6 mm inner ring)	1.1	3.0	2.2	0.0
	PD (6 mm outer ring)	0.6	2.8	1.8	3.6
	VD (6 mm circle) (mm^-1^)	1.6	2.7	0	2.2
	VD (6 mm inner ring) (mm^-1^)	1.5	4.1	1.6	1.0
	VD (6 mm outer ring) (mm^-1^)	1.1	2.1	0.54	2.7

## Discussion

Overall, the measured retinal parameters were consistent with healthy adult eyes, and longitudinal changes were consistent with normal age-related changes at the five-year follow-up. Retinal macro- and micro-vasculature were not identical between the monozygotic twins at baseline or five years later. However, our findings suggest that the retinal microvasculature and retinal layer thickness may follow heritable patterns. Conversely, differences in FAZ area and subfoveal choroidal thickness over time may particularly be more susceptible to and reflective of environmental changes in otherwise healthy individuals.

From our previous work, OCT and OCTA measurements of both subjects at both time points were within the range of measurements seen in cognitively normal controls [10]. Additionally, the velocity of change of GC-IPL thickness, RNFL thickness, SFCT, CVI, FAZ, and 6 mm VD and PD were consistent with longitudinal velocity findings in cognitively normal individuals [11]. OCT measurements also showed normal patterns of aging in the fourth decade of life in both subjects, with a less than 5% change in GC-IPL thickness, RNFL thickness, and CST, as well as a decrease in subfoveal choroidal thickness after five years, with more significant thinning anticipated after the age of 50 [12,13]. OCTA measurements were also consistent with aging, showing a decrease in macular VD [13,14].

While statistical significance cannot be commented on in this single pair of identical twins, observations on OCT are consistent with significantly heritable parameters. This is consistent with Hougaard et al.’s study, which showed that peripapillary retinal nerve fiber layer thickness had a within-pair difference of 4.6% and heritability of 66% [15]. Similarly, a study by Chamberlain et al. found that monozygotic twins had a significantly greater correlation of macular thickness [16]. The choroidal vascularity index, which is less influenced by physiologic fluctuations than subfoveal choroidal thickness measurements, demonstrated a small degree of difference (< 3% in both eyes) between the two twins at both baseline and five years later. This metric and GC-IPL thickness had the smallest measured change among almost all OCT parameters and may reflect some heritability. This is also consistent with Yoon et al.’s cross-sectional twin study of CVI, which demonstrated high heritability of CVI [17]. This supports our findings of up to a 6.2% difference in CST, GC-IPL thickness, RNFL thickness, and CVI between monozygotic twins at both baseline and five years later.

Larger differences between the twins were seen in subfoveal choroidal thickness (up to 8.1%). Although subfoveal choroidal thickness may vary with sex, refractive error, and other factors, such as smoking, exercise, time of day, and use of certain medications, none of these potentially differentiating factors differentiated either male twin [18]. This large variation in subfoveal choroidal thickness in genetically identical individuals suggests that there may not be a strong pattern of heritability with this choroidal thickness metric. This is also consistent with Sardell et al.’s study of choroidal thickness in the Amish, which showed that choroidal thickness only demonstrated moderate heritability [19]. This makes choroidal thickness a possibly strong parameter for the measurement of environmental, rather than genetic, effects.

The use of OCTA in this identical twin report allowed for analysis of the retinal microvascular capillary networks, in contrast to prior studies of healthy twins that have typically employed only conventional fundus photographs for larger retinal vessel analysis. As the microvasculature may be sensitive to different retinal stress than the larger retinal vessels, the incorporation of a multimodal approach using OCTA in larger, longitudinal studies may offer unique insights. We observed that most OCTA parameters typically differed by no more than 4.1% between these twins at both baseline and five years later. These smaller differences in genetically identical individuals that are less than 4% seen among subjects suggest that the OCTA parameters of VD and PD may potentially be heritable, although interestingly not identical in homozygotes [20]. Moreover, the stability of VD and PD over a five-year period within the twins suggests that retinal microvasculature on OCTA images may be a stronger candidate for biometric authentication than the larger retinal vessels on traditional fundus photography. Much larger differences in FAZ area were seen both within and between the twins, suggesting that FAZ area may be a stronger marker for environmental effects and may not be an ideal identifier. A large limitation of this study is the inability to comment on statistical significance. Much of the observed variation within and between subjects is within the normal test-to-test variation of OCT and OCTA, and the conversion of variable differences to percentages may capture the expected measurement error. As a result, no significant conclusions can be drawn from this data.

In this longitudinal study describing a single pair of monozygotic twins over a 5-year period, the degree of characterization in these identical twins illustrates the potential of retinal and choroidal imaging for analysis of inherited and acquired morphologies measurable by multimodal imaging parameters. Likewise, this study suggests that the retinal microvasculature is relatively stable in normal aging and may be useful in biometric authentication.

## Conclusions

This longitudinal study describing a single pair of monozygotic twins over a five-year period demonstrates that, although retinal parameters were consistent with healthy adult eyes for both twins at both time points, retinal macro- and micro-vasculature were not identical between the monozygotic twins at baseline or five years later. Specifically, retinal layer thicknesses and superficial capillary plexus vessel and perfusion densities varied little between twins, suggesting strong genetic components. However, the foveal avascular zone area and subfoveal choroidal thickness showed much larger differences and thus may be more susceptible to environmental influences.

This study provides new insights into the genetic and environmental impact on retinal imaging parameters and points to consider as we learn more about the retina as a bioidentifier. The similar, but not identical, retinal and choroidal measurements between these monozygotic twins warrant larger heritability studies of such metrics to further elucidate the interaction of genetic and environmental factors on the retinal and choroidal structure and microvasculature, which are becoming increasingly recognized as unique bioidentifiers.
